# Interactive Effect of UVR and Phosphorus on the Coastal Phytoplankton Community of the Western Mediterranean Sea: Unravelling Eco-Physiological Mechanisms

**DOI:** 10.1371/journal.pone.0142987

**Published:** 2015-11-23

**Authors:** Presentación Carrillo, Juan M. Medina-Sánchez, Guillermo Herrera, Cristina Durán, María Segovia, Dolores Cortés, Soluna Salles, Nathalie Korbee, Félix L. Figueroa, Jesús M. Mercado

**Affiliations:** 1 Instituto del Agua, Universidad de Granada, Granada, Spain; 2 Departamento de Ecología, Facultad de Ciencias, Universidad de Granada, Granada, Spain; 3 Departamento de Ecología, Facultad de Ciencias, Universidad de Málaga, Málaga, Spain; 4 Centro Oceanográfico de Málaga, Instituto Español de Oceanografía Fuengirola, Málaga, Spain; Stony Brook University, UNITED STATES

## Abstract

Some of the most important effects of global change on coastal marine systems include increasing nutrient inputs and higher levels of ultraviolet radiation (UVR, 280–400 nm), which could affect primary producers, a key trophic link to the functioning of marine food webs. However, interactive effects of both factors on the phytoplankton community have not been assessed for the Mediterranean Sea. An *in situ* factorial experiment, with two levels of ultraviolet solar radiation (UVR+PAR vs. PAR) and nutrients (control vs. P-enriched), was performed to evaluate single and UVR×P effects on metabolic, enzymatic, stoichiometric and structural phytoplanktonic variables. While most phytoplankton variables were not affected by UVR, dissolved phosphatase (APA_EX_) and algal P content increased in the presence of UVR, which was interpreted as an acclimation mechanism of algae to oligotrophic marine waters. Synergistic UVR×P interactive effects were positive on photosynthetic variables (i.e., maximal electron transport rate, ETR_max_), but negative on primary production and phytoplankton biomass because the pulse of P unmasked the inhibitory effect of UVR. This unmasking effect might be related to greater photodamage caused by an excess of electron flux after a P pulse (higher ETR_max_) without an efficient release of carbon as the mechanism to dissipate the reducing power of photosynthetic electron transport.

## Introduction

Coastal marine ecosystems contribute about 30–35% of the global production of phytoplankton in oceanic waters [1] and play a key ecological and economic role for human populations [2]. Due to the pivotal role of the marine primary productivity, any change caused by alterations in abiotic limiting factors such as light or nutrients can have consequences for the functioning of complex marine food webs [2,3]. Some of the most important effects of global change on coastal marine systems include increasing input of nitrate and phosphate, brought about by both climate change (e.g. atmospheric deposition, wind, precipitation) and increased land use (e.g. agricultural activities [4]) causing eutrophication [5].

In addition, phytoplankton might be exposed to higher levels of solar ultraviolet radiation (UVR, 280–400 nm) because of both the greater stratification of the water column due to global warming [6,7,8] as well as the depletion of the ozone layer [9] or low ozone episodes (ozone mini-hole), which imply UVR short and variable intensity pulses, (i.e. 1–5 days [10], as occurs in Mediterranean coastal areas [11]. It has also been reported that at some sites of the Northern Hemisphere, UVB irradiance may continue increasing because of continuous reduction in aerosol extinction since 1990 [12]. Reductions in aerosols and clouds are expected to overcompensate for the effect of ozone recovery UVR after the Montreal protocol. Therefore, UVR still remains as a world-wide stressor with far-reaching implications for ecological interactions [13]. UVR causes damage to the cells directly, via the photochemical degradation of biomolecules (e.g. nucleic acids, lipids, proteins) [14], or indirectly, via the production of reactive oxygen species (ROS) generated by chemical, photolytic reactions inside and outside the cells [15,16]. The negative effects of UVR at the sub-cellular level could lead to reductions in productivity and growth rates at the phytoplankton community level [17,18,19]. However, previous studies also have evidenced a null [20] or positive [21] effect of UVR on phytoplankton. Different protective strategies such as improved efficiency of repair processes at the sub-cellular level of algae exposed to high UVR [22], synthesis of photoprotective compounds [23,24] or changes in taxonomic composition towards communities better adapted to the new conditions [25] are among the mechanisms evolved to allow the acclimation of algae to UVR in marine waters.

The mechanisms that are best suited for adaptation to UVR depend on cell-nutrient content [13]. The direct effects of UVR-inorganic nutrient interactions on phytoplankton can be twofold: firstly, UVR affects nutrient uptake and/or assimilation capacities of the phytoplankton, which is often a UVR species-specific response [26]. Nevertheless, the interaction of UVR with P acquisition mechanism is likely to reflect additional complexities related to biogeochemical processes and/or biotic interactions. Access to dissolved organic phosphorus (DOP) can be increased by alkaline phosphatase (APA), which catalyses the hydrolysis of phosphate esters into orthophosphate [27]. This extracellular enzyme may play a major role in P supply in P-deficient ecosystems, in which phosphate concentrations are usually negligible and most of the P is bound to organic matter [28]. There is evidence for both inhibition [29] and stimulation [30,31] of APA by UVR. Secondly, the nutrient status of phytoplankton may determine the susceptibility of these organisms to UVR [32]. Despite that nutrient-deficient phytoplankton, especially those limited by N [6,25,33], are generally considered to be more sensitive to UVR than are nutrient-replete phytoplankton, some authors have reported a higher sensitivity of phytoplankton to UVR under nutrient-replete conditions [34]. Variations in the type of nutrient that limits phytoplanktonic growth or the severity of this limitation, as well as differential tolerance/response to UVR by the phytoplanktonic community [26], are some of the potential interpretations proposed to explain the wide variability of algal responses to UVR and nutrients.

Moreover, it has been shown that, together with the nutrient content in the phytoplanktonic cells, their elemental composition (C:P and N:P ratios) plays a key role in determining the algal response to UVR. Along this line, several studies have demonstrated a direct effect of UVR, decreasing elemental C:P in marine and freshwater phytoplankton [20,31,35,36]. Due to the relevance for C and P cycling in the marine ecosystems, it is of interest whether C:P under UVR exposure relate primarily to metabolic features such as lower C-fixation, greater C-losses by respiration or organic carbon release, and/or whether the exposed cells also have the capability of improving their P-content via a stimulatory UVR effect on APA activity [31].

The literature addressing UVR effects on inorganic nutrient cycling has focused mainly on N [37,38,24], as this element has generally been reported to limit primary production in marine environments. Less is known about interactions between UVR and P utilization and the phytoplanktonic metabolism in marine ecosystems. The Mediterranean Sea occupies an oligotrophic basin where primary production is recognized to be limited by P [39,40,41], although more-recent reports have modified this paradigm [42]. Several studies on nutrient enrichment have shown that plankton growth is often stimulated after P addition in surface waters during the stratification period [43,44,45,46].

Information about the interactive effect of UVR and P in oligotrophic marine ecosystems is noticeably lacking. This topic is raising great interest within the current global-change scenario, where inorganic inputs from terrestrial origin linked to human activity or due to increasing intensity and frequency in atmospheric dust inputs rich in P from the Sahara Desert is reaching the Mediterranean Sea [47,48] (http://ozoneaq.gsfc.nasa.gov/).

The aim of the present study was to quantify single and combined effects of UVR flux and changes in the P supply on primary producers, paying special attention to the physiological mechanisms involved in the responses of the phytoplankton communities. Our hypothesis is that single effects of UVR and P-addition will be inhibitory and stimulatory, respectively, on marine phytoplankton, thereby producing an antagonistic interactive effect consisting of the attenuation of inhibitory UVR effects by P addition. For this purpose, an *in situ* experiment was conducted in oligotrophic clear waters at Cabo de Gata, on the Alborán Sea, Spain. Light quality and nutrient (P) availability were manipulated at a medium-term scale since the phytoplankton response to these nutrient-enrichment episodes is quite rapid (24–48 h) and phytoplankton bloom decline within 5–6 days after reaching the biomass peak [49,50].

## Material and Methods

### Study site

**Fig 1:**
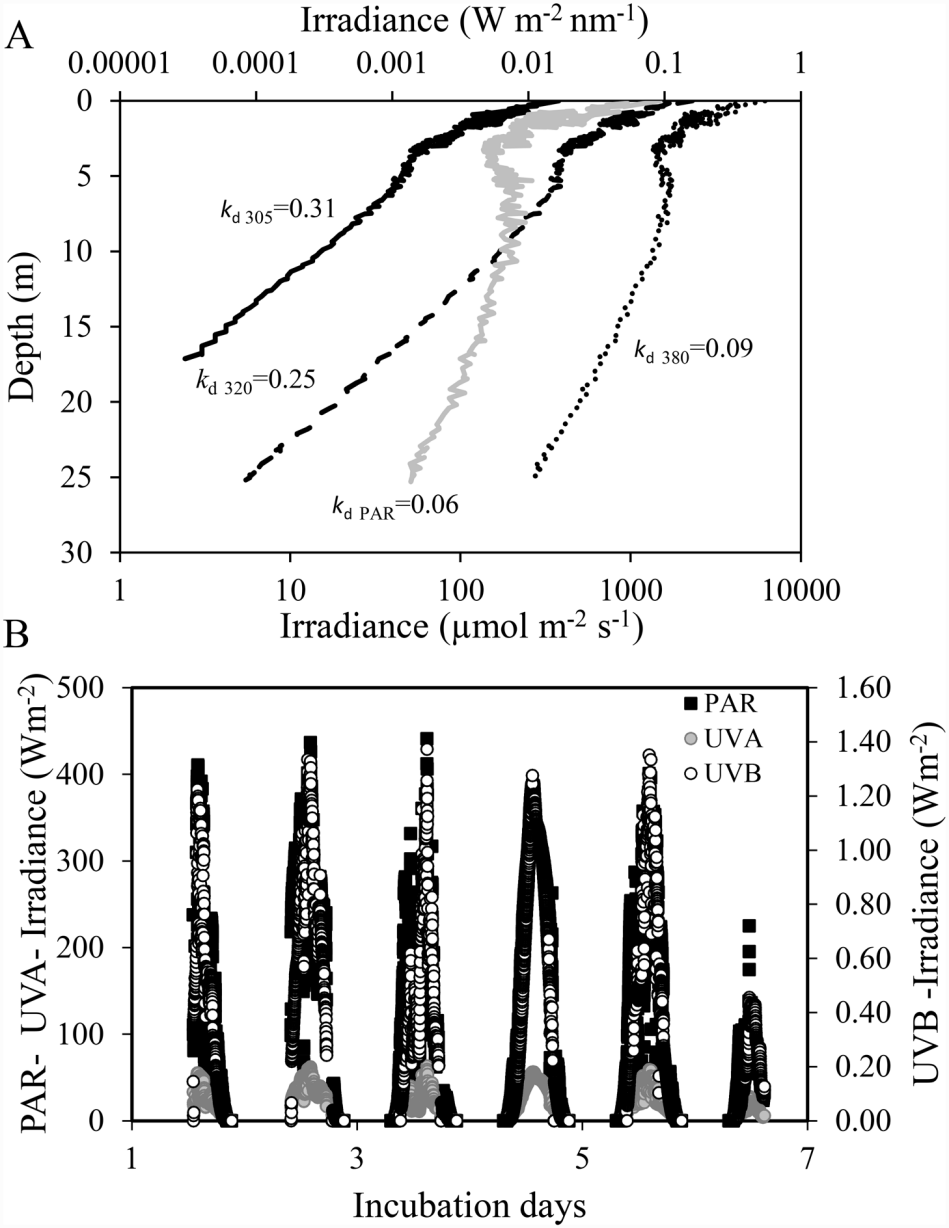
Solar radiation during the experiments in Cabo de Gata. (A) Depth profiles of the irradiance at 305, 320, and 380 nm, and PAR (400–700 nm) and values of vertical attenuation coefficients (m^-1^); (B) Solar radiation reaching the deck of the boat during the experiments. Solar radiation was continuously monitored using a broad-band filter radiometer instrument (ELDONET, Real Time Computer, Möhrendorf, Germany) placed on the deck.

Water samples for the experiments were collected on board B/O F. de P.Navarro (Spanish Oceanography Institute) offshore of Cabo de Gata Natural Park (36° 33’N, 2° 16’W), located on the eastern end of the Alborán Sea. The sampling was performed during summer, when the water column was characterized by a strong thermal stratification, leading to an impoverishment of nutrients in the euphotic layer. Cabo de Gata is bio-optically characterized by very clear waters, resulting in very low values of diffuse attenuation coefficients for downward irradiance and high surface irradiances (Fig 1A). No specific permissions were required to conduct our experiments on microplanktonic communities due to the low impact on area of study. Our research involve no endangered or protected species.

### Experimental set-up

The experiment consisted of a factorial design with two light treatments [full sunlight (UVR+PAR; 280–700 nm) and only photosynthetic active radiation (PAR; PAR;400-700nm)] and two phosphorus (P) treatments [increased P (P-enriched treatment) and ambient nutrient conditions (control treatment)]. Water samples were collected with Niskin bottles at different depths within the upper 10-m layer at the sampling station (photic layer receiving > 1% surface UVR_305_). The bottle water was pooled and mixed in order to fill a 240-L tank. Water was pre-screened through a 200-μm nylon mesh to remove mesozooplankton. Mesozooplankton was excluded because the volume of the microcosms was not suitable to maintain a grazer community for a week, and therefore we avoided the lack of replicability in the microcosms due to the uneven effect of herbivores on phytoplankton. Tank water was used to fill twelve 20-L low-density polyethylene (LPDE) (Plásticos Andalucía, Spain) microcosms in triplicate. LPDE transmits 90% of photosynthetic active radiation, 75% of UVA and 60% of UVB).

Microcosms were incubated from 21 to 26 September 2009 in two tanks on deck of the boat to avoid possible damage underwater. To ensure natural light conditions, we painted the two tanks black, thereby avoiding background reflection. Temperature was kept constant by continuously pumping surface seawater. For the UVR-exclusion treatment, the tank was covered with a polycarbonate filter of UF3 plexiglass (Atohaas, USA) which screens out UVR < 390nm and transmits 90% of the PAR. For the P-enrichment treatments, KH_2_PO_4_ was added to half of the microcosms to a final concentration of 30μg P L^-1^, which are the estimated P inputs from Saharan dust to the Mediterranean area [51]. Different samples of each microcosm were collected on the sixth incubation day in order to measure different chemical, biological, and physiological variables.

### Physical measurements

Downwelling irradiances for the bands 305, 320, and 380 nm, and PAR as well as temperature were measured at different depths within the water column at noon by using a submersible BIC Compact 4-Channel Radiometer (Biospherical Instruments Inc. CA, USA). Diffuse attenuation coefficients for downward irradiance (k_d_) were calculated from the slope of the linear regression of the natural logarithm of downwelling irradiance vs. depth for the different radiation bands. Solar radiation received by the microcosms at the surface was continuously monitored using a broad-band filter radiometer instrument (ELDONET, Real Time Computer, Möhrendorf, Germany) placed on the boat deck.

### Chemical and stoichiometric analyses

Samples for total and dissolved inorganic nutrient (N and P) were frozen at -30°C until analysed. Nitrate, nitrite, phosphate, and silicate were measured by a Technicon Auto Analyzer (TrAAcs 800, Bran-Leubbe) following [52] Ramírez et al. (2005). For determination of total nitrogen (TN) and total phosphorus (TP), the samples were treated with an oxidizing agent (a mixture of peroxysulphate and boric acid in NaOH) and autoclaved for 30–40 min. Nitrate and phosphate concentrations in these samples were determined by following the methods described by Grasshoff et al. [53].

Sestonic elemental composition and stoichiometric ratios were determined from 500-mL aliquots of sample water filtered through pre-combusted (1h at 550°C) 0.7-μm filters (Whatman GF/F) at low pressure (< 100mmHg). Filters for P, N, and C were immediately frozen at -20°C. C and N analyses were performed using a Perkin-Elmer 2400 elemental analyser. P filters were introduced in acid-washed vials and digested with potassium persulfate and boric acid at 120°C for 30 min. Particulate P was then determined as SRP in 10-cm quartz cuvettes by the acid molybdate technique [53]. C:P, N:P and C:N ratios were calculated on a molar basis. Dissolved organic carbon (DOC) was measured using a total organic carbon analyser (TOC-V CSH/CSN, Shimadzu) in samples pre-filtered through pre-combusted (2 h at 500°C) glass-fiber filters (Whatman GF⁄F and acidified with HCl 1N (2%).

### Analyses of functional and structural variables

#### Alkaline phosphatase activity

APA was determined following [54] total and extracellular APA (APA_T_ and PA_EX_) were determined from unfiltered water sample from the microcosms (< 200 μm mesh) and samples filtered through 0.2-μm filters (Anodisc, Whatman) respectively. APA was calculated in nMP h^-1^ from a reference curve constructed using different concentrations of 4-methylumbelliferyl phosphate hydrolysed to methylumbelliferone (MUF, Sigma-Aldrich) and measured fluorometrically (λ_em_365nm and λ_ex_440nm)(Perkin-Elmer LS 55 luminescence-spectrometer) after 45 min. Particulate APA was calculated as the difference between APA_T_ and APA_EX_.

#### Respiration rates

Two *25*-mL samples from each microcosm were used to measure the total microplanktonic respiration (TMR) and respiration of the fraction < 0.7 μm (R _< 0.7μm_). It was estimated from oxygen depletion in darkness measured with sensor-spot optodes (SP-PSt3-NAU-D5-YOP and Fibox3; PreSens GmbH, Germany). Optodes followed a two-point calibration (0 and 100% oxygen saturation). Zero % point calibration was performed by adding sodium sulphite (Na_2_SO_3_) to a final concentration exceeding 0.1mg mL^-1^, and 100% by inserting wet cotton wool into the closed flask to ensure 100% O_2_-saturated water-vapour air. Flasks were incubated under dark conditions and constant ambient temperature (20°C) in culture chambers for 24 h and measured every 2h after 30 min of acclimation. Oxygen data were then adjusted to a linear model via least-squares regression. The slope of the regressions provided the oxygen-consumption rates. Respiration of phytoplankton was calculated as the difference between TMR and R _< 0.7μm_.

#### Reactive oxygen species

The intracellular accumulation of reactive oxygen species was estimated by using a modification of the method used by Segovia and Berges [55] described elsewhere [15]. Briefly,ROS was detected using carboxy-H_2_DFFDA (Invitrogen, Oregon, USA). Cells were incubated with 5μM c-H_2_DFFDA (final concentration) at 16°C for 90 min in darkness. Fluorescence was detected using a microplate fluorescence reader (FL-600, BIO-TEK) at an excitation of 490 nm and emission of 525 nm. The concentration of ROS in the cells was then expressed as relative fluorescence units per cell.

#### Photosynthetic rate as *in vivo* Chlorophyll Fluorescence

*In vivo* chlorophyll fluorescence was determined by using a Water-PAM pulse amplitude modulated fluorometer (Walz GmbH, Effeltrich, Germany). First, 1.5 mL of water from each microcosm was placed into the 15-mm-diameter quartz cuvette inserted in the measuring chamber. After a 15-min dark-adaptation period followed by a 5-s far-red (FR) light pulse to ensure full oxidation of Q_A_, a rapid light curve (RLC) program (WINCONTROL software, Walz GmbH) was initiated. RLCs were constructed from a 20-s exposure to each of 11 incremental irradiances (30, 46, 69, 104,158, 230, 350, 520,737, 1017, and 1642, μmol photons·m^-2^·s^-1^).

RLCs were constructed by calculating the electron-transport rate (ETR) through PSII for each level of actinic light:

$$ETR=\left(\frac{F{´}_{m}-F}{F{´}_{m}}\right)*\text{AQ }\cdot \text{ FII}$$ (1)

where (F’m-F)/F’m estimates the effective quantum yield of PSII, and AQ is the absorbed quantum expressed as *μ*mol m^-2^ s^-1^, calculated as follows:

$$\text{AQ}=\sum_{400}^{700}{\text{a}}_{{\text{ph}}_{}}(\lambda )\times \text{E}(\lambda )$$ (2)

The specific absorption coefficient (a_ph_ (*λ*), expressed as m^-1^) was estimated by calculating the light absorption by phytoplankton concentrated on glass-fiber filters corrected as described by Korbee [56]. E(*λ*) is the spectral irradiance of the LED lamp of the Water-PAM and F_II_ is a multiplication factor (adimensional) that expresses the fraction of absorbed quanta to PS_II_, between 400–700 nm. This value was calculated for each treatment as a function of the percentages of bacillariophyceae, dinophyceae I, and flagellates, taking into account the F_II_ values proposed by Johnsen and Sakshaug [57] for the different groups. In all cases, we used the values for the high-light-acclimated cultures, as these organisms, being cultured at the surface, were exposed to high irradiances.RLC data were fitted to the model of Eilers and Peeters [58] to calculate values for the initial slope (α_ETR_) and maximal electron transport rate (ETR_max_). The light-saturation parameter (E_k_) was derived from ETR_max_ and ɑ_ETR_:

$$E_{k}=\frac{ETR_{max}}{\alpha }$$ (3)

#### Photosynthetic pigments and xanthophyll cycle

A 300-mL sample of water from each microcosm was filtered through glass fibre (Whatman GF/F filters) at < 100mmHg. Filters were preserved at -80°C until analysed. The filters were immersed in N, N-dimethylformamide and extracted overnight at 4°C. Pigment concentration in the extract was determined by HPLC after filtration through 0.2 μm following Lubian and Montero [59]. Identification and quantification were made using commercial standard (DHI LAB products). Concentrations of chlorophyll a (Chl *a*), chlorophyll c2 (Chl c2), diadianoxanthin (Dd), diatoxanthin (Dt), fucoxanthin, zeaxanthin, and traces of other xanthophyll pigments were detected.The Dd de-epoxidation degree (in %) was calculated as follows:

$$Dd\;de-epoxidation=\frac{Dt}{Dd+Dt}$$ (4)

#### Primary production and excreted organic carbon

From each microcosm, 200 mL were used for primary production (PP) measurements following the ^14^C method proposed by Steeman-Nielsen [60]. For this, 9.25 MBq of NaH^14^CO_3_ (specific activity: 310.8 MBq mmol^-1^, DHI Water and Environment, Germany) were added to sets of four 50-mL flasks (three clear and one dark as control). The flasks were incubated in the tanks at the same depth as the microcosms, for 4 h symmetrically distributed around noon. The total organic carbon (TOC) produced was measured on 4-mL aliquots before filtration. Microplankton primary production (PP_M_) was determined from particles retained by filtering the sample through 3-μm glass-fiber filters (Whatman GF/D). The filtered water was used for picoplanktonic primary production (PP_P_) in a subsequent filtration and estimated from particles retained in 0.7 μm (Whatman GF/F). Filtrations at low pressure (< 100 mmHg) were used to minimize cell breakage. The excreted organic carbon (EOC) was measured from 4-mL aliquots of the < 0.7-μm filtrate. Filters and filtrate were placed in 20-mL scintillation vials and acidified with 100 μL of 1N HCl to remove DI14C. Vials were then kept open for 24 h in an aeration hood following the recommendations of Lignell [61]. Sixteen mL of scintillation cocktail (Ecoscint A) were added for scintillating the vials and counted using a scintillation counter (Beckman LS 6000TA) equipped with autocalibration. The %EOC was estimated as the percentage of EOC to TOC:

$$\%EOC=\frac{EOC}{TOC}\times 100$$ (5)

#### Abundance/biomass and taxonomical composition of phytoplankton

Water samples of 200 mL were taken for taxonomical identification and quantification of autotrophic microplankton ˃ 5 μm) by means of inverted microscope. The sample was preserved in acetic lugol solution and cell abundance was determined by the Utermöhl technique. Also, 5 mL of sample were fixed with glutaraldehyde in order to quantify cell abundance of autotrophic picoplankton (AP; *Prochlorococcus*, *Synechococcus* and pico-eukaryotes with a Becton Dickinson FACScan flow cytometer; more details in [62]). Biovolumes of the three pico-phytoplankton cell groups analysedwere calculated following Ribes et al. [63] for samples collected in the north-western Mediterranean Sea. Biovolume values were converted into biomass by using the formulae proposed by Morel et al. [64] for *Prochlorococcus* and Kana and Glibert [65] for *Synechococcus*. Cell biovolumes corresponding to the most abundant species, genus, and other taxa of microphytoplankton identified by inverted microscope were calculated by using the appropriate formula according to their geometric shape [66, 67, 68]. Biovolumes were converted into biomass using the formulae proposed by Verity et al. [69] for phytoplankton < 15 μm and Menden-Deuder and Lessard [70] for ˃15 μm dinophycecae and bacillariophyceae.

### Statistical analysis

A full factorial two-way ANOVA test was used to evaluate the interactive effects of light and nutrients on all variables after normality (Kolmogorov-Smirnov test) and homoscedasticity (Cochran C test) were verified. When interactive effects of both factors were found, a *post hoc* Tukey HSD test was used to account for significant differences between treatments. All tests were performed using R2.15 software (R Development Core Team).

## Results

### Starting conditions of the experiments

Changes with depth of the downwelling irradiance of the different spectral bands as well as surface solar radiation during the experimental period are shown in Fig 1A and 1B. There was a weak at tenuation of UVR in the water column, since UVB and UVA spectral bands reached 13 and 25 m in depth, respectively (Fig 1A). The penetration depth of PAR was greater, although the attenuation coefficient (k_PAR_) was also relatively low (0.075 m^−1^). Surface UVR and PAR irradiance (Fig 1B) proved similar during the first five days of the experiment, reaching maximal values of ca. 70 and 450 W m^−2^ for UVR and PAR, respectively. By contrast, the last day of experimentation was cloudy and consequently the maximal irradiance was comparatively lower. The daily mean irradiance values during the experimental period (i.e. total duration of exposure of microcosms) for UVB, UVA, and PAR were 0.14, 18.72 and 135.88 W m^−2^, respectively. The concentrations of total and inorganic forms of N and P indicate that most of the nutrients were in particulate form incorporated into the organisms. The DIN:TP ratio was low, implying likelihood of N limitation for the pelagic community, although the sestonic N:P ratio was close to the Redfield ratio (Table 1). Chl *a* concentrations were < 1 μg L^-1^, this being characteristic of oligotrophic ecosystems.

**Table 1:** Physical, chemical, and biological variables at initial conditions of the experiment in Cabo de Gata. Values are mean and standard deviation (sd)Variables.

Physical, chemical, and biological variables at initial conditions of the experiment in Cabo de Gata	Mean values ± sd
T (°C)	21.1±1.20
SRP (μM)	0.10 ±0.06
TN (μM)	4.12 ±0.60
TP (μM)	1.50 ±0.45
DIN (NO_3_+NO_2_^-^)(μM)	0.41±0.01
DIN/TP (molar)	0.27±0.05
Si (μM)	0.24± 0.012
Chl *a* (μg L^-1^)	0.99±0.23
Total APA (nM P h^-1^)	8.57±1.27
PP_M_ (μg C·L^-1^)	2.37±0.88
PP_P_ (μg C·L^-1^)	0.62±0.25
POC (μM)	26.43±3.04
PON (μM)	3.09±0.39
POP (μM)	0.15±0.02
C:N ratio	8.57±0.17
N:P ratio	20.30± 4.79
C:P ratio	173.49 ±38.62
DOC (mgL^-1^)	1.72±0.23
PA (cell·mL^-1^)	7,895±1,489
PB (μg C·L^-1^)	172.14±32.20

Abbreviations: T = temperature; SRP = soluble reactive phosphorus; TN = total nitrogen; TP = total phosphorus; DIN (NO_3_^-^+NO_2_^-^) = dissolved inorganic nitrogen (nitrate + nitrite); Si = silicates; Chl a = chlorophyll a; APA = total alkaline phosphatase activity; PP_M_ = microplanktonic primary production; PP_P_ = picoplanktonic primary production; POC = particulate organic carbon; PON = particulate organic nitrogen; POP = particulate organic phosphorus; DOC = dissolved organic carbon; PA = phytoplanktonic abundance; PB = phytoplanktonic biomass. ɤAll measurements were made on the mixed water simple, taken from the photic layer receiving>1% surface UVR_305_

Total initial phytoplankton was ~7.8 10^3^ cells mL^−1^ and 172.14 μgC L ^-1^, in term of abundance and biomass, respectively (Table 1). Diatoms represented 58% total biomass, followed by flagellates (26%). Picoplankton made up 14% of the total biomass (Table 2), although the dominant group in terms of abundance was S*ynechococcus* sp, which accounted for 33%and picoeukaryotes 44% of total abundance

**Table 2:** Changes in the taxonomical composition of phytoplankton communities during the experiment. Phytoplanktonic groups or species biomass (μg C L^-1^) and percentage of biomass (%) under initial conditions and after each treatment. Values are mean and standard deviation whereas the letters indicate differences among treatments for the different phytoplanktonic groups and total biomass.

			CONTROL				P-ENRICHED			
	INITIAL		UVR+PAR		PAR		UVR+PAR		PAR+P	
	Mean ±sd	%	Mean ±sd	%	Mean ± sd	%	Mean ± sd	%	Mean ± sd	%
**Bacillariophyceae**	100.46±16.13	58.36	205.74±23.27 ^a^	57.73	175.42±62.81 ^a^	52.36	136.24±26.13 ^a^	68.95	157.98±35.95 ^a^	47.90
**Dinophyceae**	1.65±0.96	0.01	2.94±1.09 ^a^	0.01	2.02±1.00 ^a^	0.01	2.32±2.16 ^a^	0.01	1.32±0.46 ^a^	0.00
**Flagellates**	44.89±0.87	26.07	146.45±34.39 ^a^	41.10	156.15±36.66 ^a^	46.61	52.47±13.54 ^b^	26.56	156.54±26.84 ^a^	47.47
**Picoeukaryotes**	22.31±0.45	12.96	0.85±0.19 ^a^	0.24	0.74 ± 0.07 ^a^	0.22	5.22±0.25 ^b^	2.64	11.94±0.50 ^c^	3.62
***Prochlorococcus* sp.**	0.004±0.001	0.00	0.003±0.002 ^a^	0.00	0.001 ± 0.0003 ^a^	0.00	0.002±0.0003 ^a^	0.00	0.002±0.001 ^a^	0.00
***Synechococcus* sp.**	2.83±0.08	1.64	0.37±0.02 ^a^	0.11	0.68 ±0.02 ^b^	0.20	1.34±0.12 ^c^	0.68	2.02±0.12 ^d^	0.61
**Total biomass**	172.14±32.39		356.37±5 9.31 ^a^		335.02 ±99.45 ^a^		197.61±41.24 ^b^		329.80±62.43^a^	

### UVR and P effect on ROS, R, APA, DOC, and C:N:P ratio

**Fig 2:**
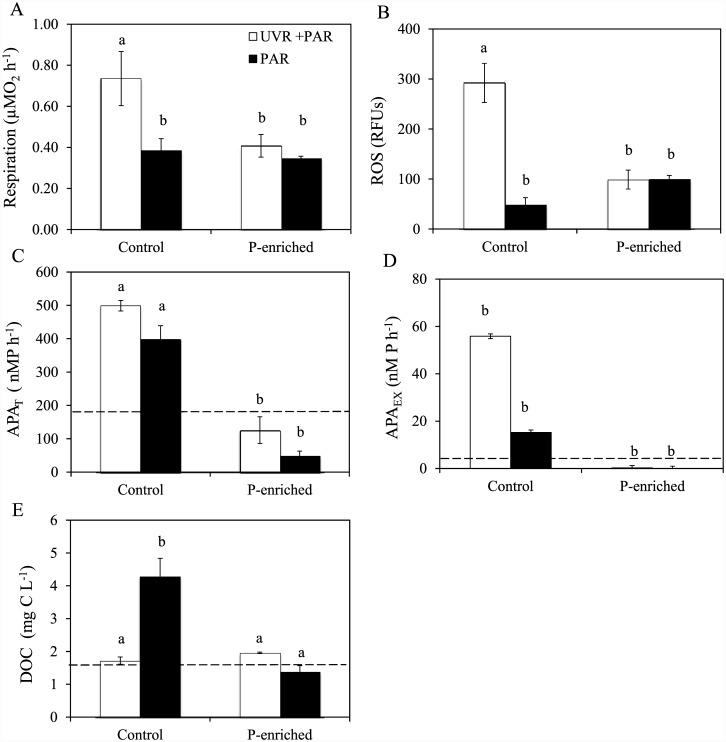
Response of metabolic, enzymatic variables and dissolved organic carbon to experimental conditions. (A) Respiration rates (R, in μMO_2_ h^-1^); (B) Reactive oxygen species (ROS, in RFUs); (C) total alkaline phosphatase (APA_T_ in nMP h^-1^); (D) Dissolved alkaline phosphatase (APA_EX_ in nMP h^-1^); (E) Dissolved organic carbon (DOC in mgC L^-1^) under photosynthetically active radiation (PAR), and full sunlight (PAR+UVR) in nutrient-enriched (P-enriched) and non-enriched (control) treatments. Horizontal dashed lines indicate value for initial day. Data are expressed as mean values ± sd (n = 3). Significant differences among treatments are denoted by lower-case letters.

Under non-enriched conditions, UVR significantly augmented R and ROS values (Fig 2A and 2B). P enrichment eliminated this increasing effect of UVR, generating a significant UVR×P effect (Table A in S1 File), which, according to our hypothesis, reduced the UVR-stress effect on both variables. Similarly, UVR alone had a significant effect on APA_T_ and APA_EX_ (Fig 2C and 2D), increasing APA activities by 25 and 265% respectively, but not on particulate APA (data not shown). As expected, P-enrichment significantly reduced APA activities in both fractions and cancelled the stimulatory UVR effect on APA_T_ and APA_EX_, although no significant UVR×P effect on them was found (Table A in S1 File). DOC, in non-enriched treatments, decreased (60%) under UVR compared to PAR (Fig 2E). After P enrichment, DOC decreased in relation to PAR control treatment in both light treatments that generated a significant interactive UVR×P effect on this variable (Fig 2E, Table A in S1 File).

**Fig 3:**
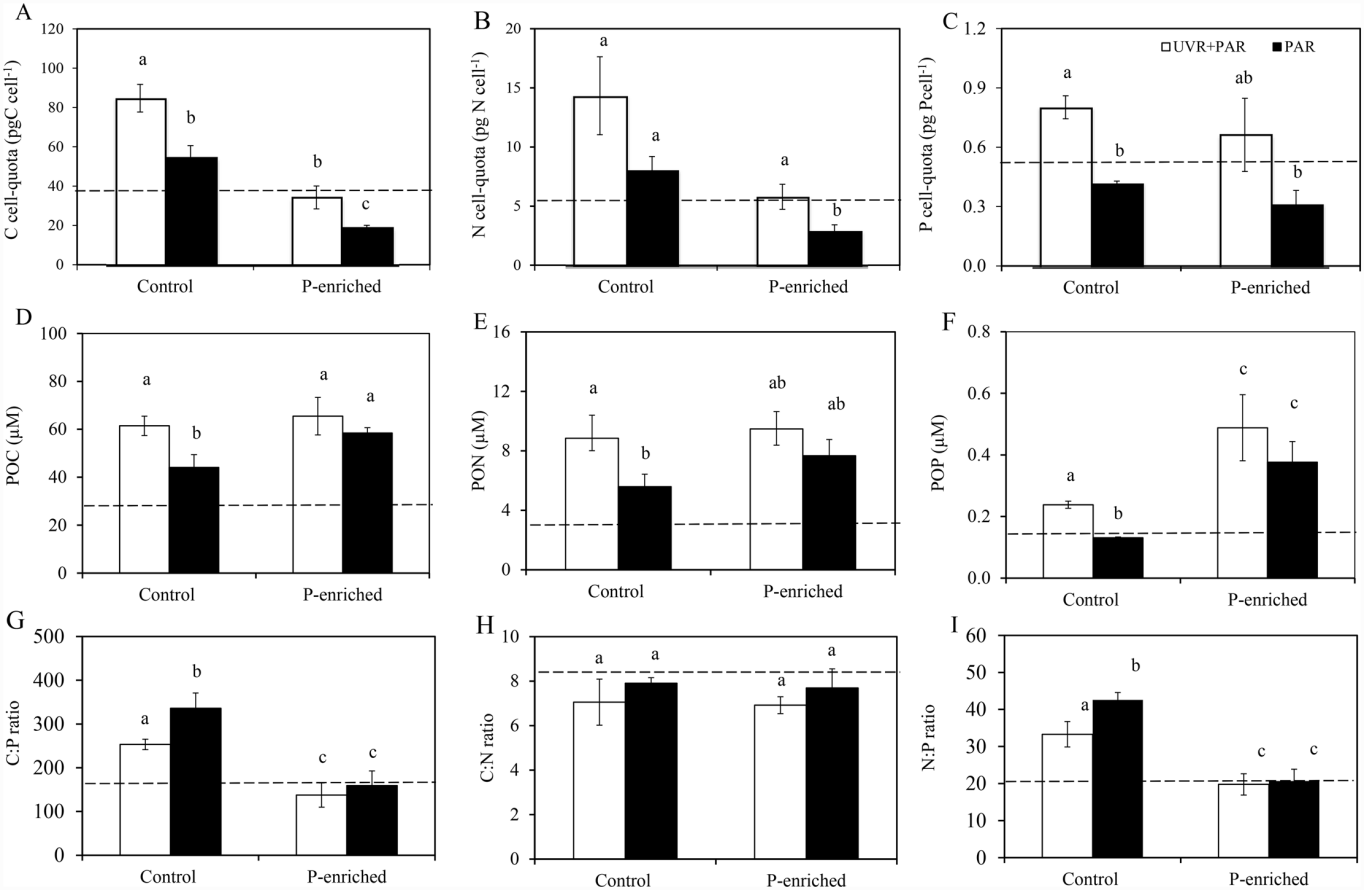
Elemental content of the sestonic fraction under experimental conditions. (A) C cell-quota (pgC cell^-1^); (B) N cell-quota (pgN cell^-1^); (C) P cell-quota (pgP cell^-1^); (D)particulate organic carbon (POC; μgC L^-1^); (E) Particulate organic nitrogen (PON; μgN L^-1^); (F) Particulate organic phosphorus (POP; μgP L^-1^); (G) C:P; (H) C:N and (I) N:P ratios under photosynthetically active radiation (PAR) and full sunlight (PAR+UVR) in nutrient-enriched (P-enriched) and non-enriched (control) treatments. Horizontal dashed lines indicate value for initial day. Data are expressed as mean values ± sd (n = 3). Significant differences among treatments are denoted by lower-case letters.

Noticeably, under non-enriched conditions, C, N, and P cell-quota and POC,PON, and POP increased significantly under UVR (Fig 3A–3F). The relative increase of POP was higher in the UVR (60%) than in the PAR treatment, resulting in a significant decline in C:P molar ratio (25%) (Fig 3G). P enrichment significantly diminished values of C-, N-but not P cell-quota (Fig 3C). By contrast, P enrichment resulted in a significant increasing effect only on POP, and therefore C:P (~140) and N:P (~20) decreased regardless of light treatments (Fig 3G and 3I; Table B in S1 File). The C:N ratio (mean values 7.40) did not change among treatments.

### UVR and P effect on photosynthetic activity, pigments, and primary production

**Fig 4:**
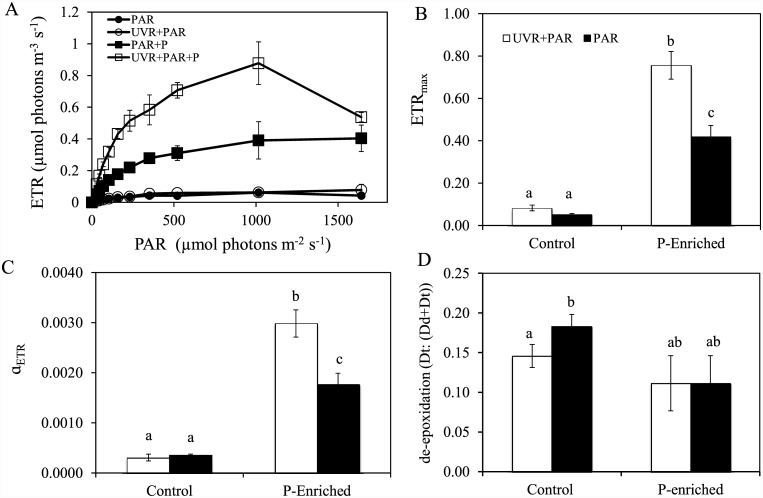
Response of photosynthetic rate and xanthophyll cycle pigments to experimental conditions. (A) The electron-transport rate (ETR) vs. irradiance; (B) ETR_max_; (C) Initial slopes (α_ETR_) of curves ETR; (D) Dd de-epoxidation state of the xanthophyll cycle pigments (D_t_:(D_t_+D_d_) (diadinoxanthin, D_d_, and diatoxanthin, D_t_) under photosynthetically active radiation (PAR) and full sunlight (PAR+UVR) in nutrient enriched (P-enriched) and non-enriched (control) treatments. Data are expressed as mean values ± sd (n = 3). Significant differences among treatments are denoted by lower-case letters.

Under non-enriched conditions, UVR did not affect ETR_max_ or F_v_/F_m_ (data not shown). However, ETR_max_ and photosynthetic efficiency (α_ETR_) were higher in the UVR and P-enriched treatment (Fig 4A, 4B and 4C). Hence, the UVR×P interaction was synergistically positive on ETR_max_ and (α_ETR_) (Table C in S1 File). In general, chlorophyll and carotenoid pigments (fucoxanthin) did not respond significantly to any treatment assayed (data not shown), and only the D_t_:(D_d_+D_t_) ratio decreased under UVR at ambient nutrient conditions (Fig 4D; Table C in S1 File).

**Fig 5:**
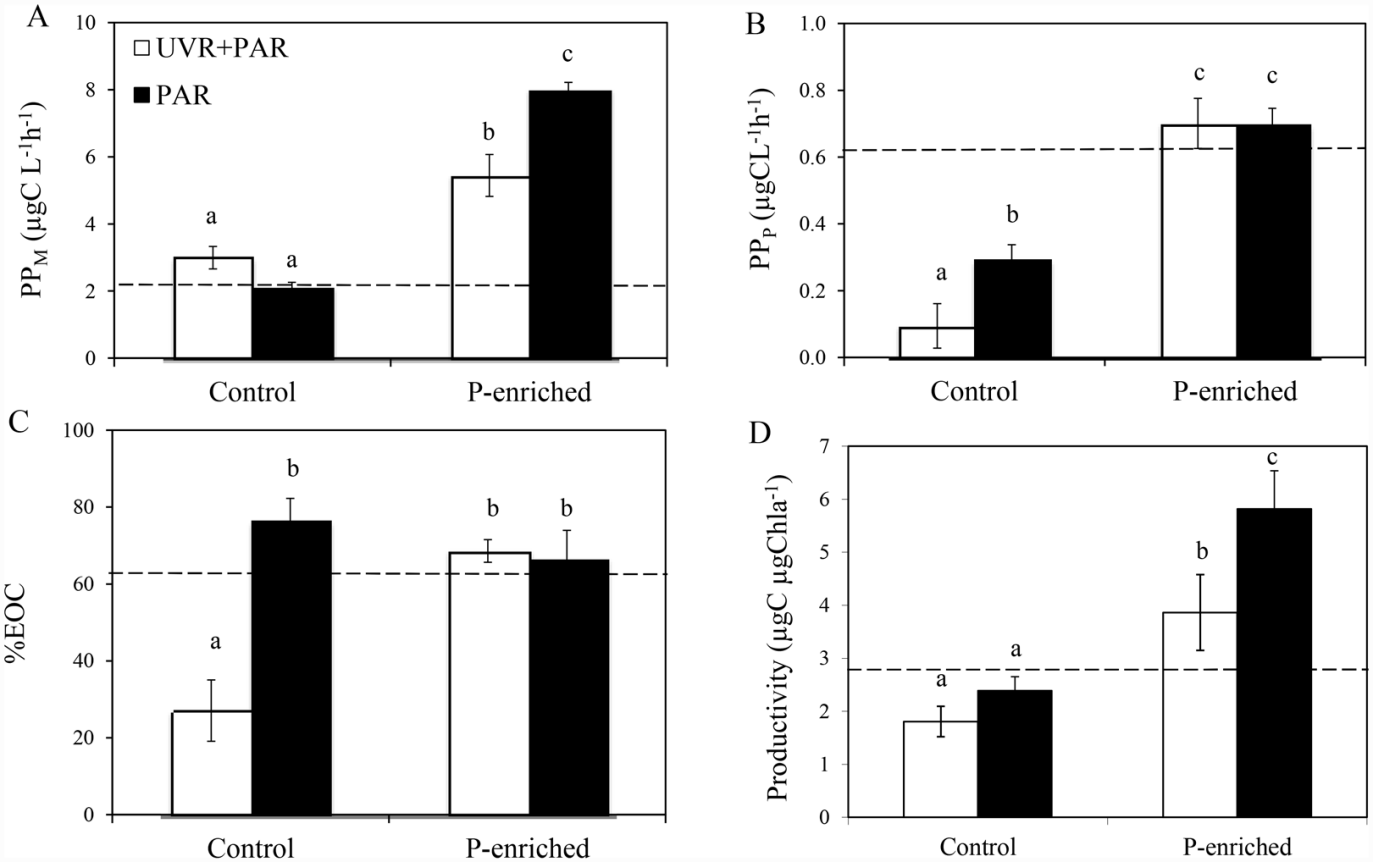
Primary production in different size fractions, excreted organic carbon (%) and productivity under experimental conditions. (A) Microplanktonic primary production (PP_M_,in μgC L^-1^ h^-1^); (B) Picoplanktonic primary production (PP_P_, in μgC L^-1^ h^-1^); (C) Excreted organic carbon (% EOC); (D) Productivity (μgC μgChl *a* ^-1^) under photosynthetically active radiation (PAR) and full sunlight (PAR+ UVR) in nutrient-enriched (P-enriched) and non-enriched (control) treatments. Horizontal dashed lines indicate value for the initial day. Data are expressed as mean values ± sd (n = 3). Significant differences among treatments are denoted by lower-case letters.

PP_M_ represented more than 88% of the total PP. The effect of UVR on PP varied according to the phytoplankton size fraction being considered (Fig 5A and 5B). Thus, under non-enriched conditions PP_M_ was not affected by UVR but PPP decreased (up to 68%; Fig 5B). P-enrichment had a stimulatory effect on both size fractions, unmasking a negative UVR effect on PP_M_ and productivity, but suppressing the inhibitory UVR effect on PP_P_ (antagonistic effect). EOC rates (data not shown) and %EOC had significantly the lowest values in samples under UVR under non-enriched conditions whereas P-enrichment increased both variables, generating an antagonistic UVR×P effect (i.e. joint UVR and P eliminated the decreasing UVR effect on the EOC rate (data not shown) and %EOC (Fig 5C).

### UVR and P effect on abundance/biomass and taxonomic composition of the phytoplankton

**Fig 6:**
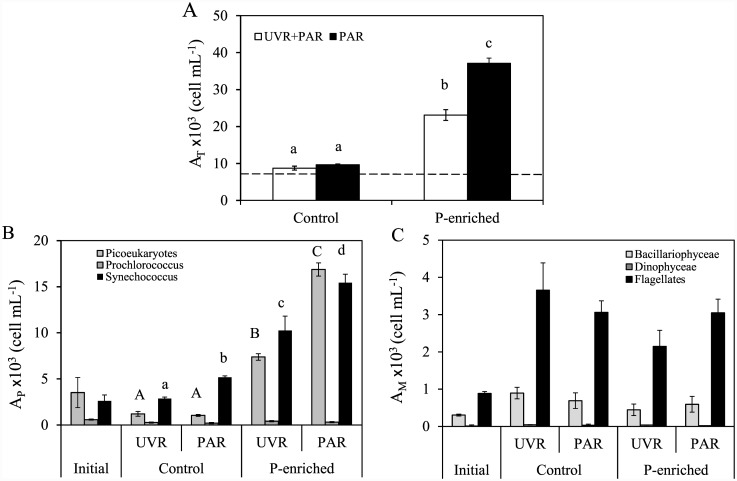
Phytoplanktonic abundance under experimental conditions. (A) Total phytoplankton abundance (AT) (cells mL^−1^); (B) Picoplanktonic abundance (A_P_) in cells mL^−1^); (C) Microplanktonic abundance (A_M_ in cells mL^−1^) under photosynthetically active radiation (PAR) and full sunlight (PAR+UVR) in nutrient-enriched (P-enriched) and non-enriched (control) treatments. Data are expressed as mean values ± sd (n = 3). Significant differences among treatments are denoted by lower-case (total abundance and *Synechococcus* sp. abundance) and capital letters (picoeukaryotes abundance).

Total phytoplankton abundance and biomass, in non-enriched treatments, showed no significant differences between light treatments (Fig 6A; Table 2). Total phytoplankton abundance increased in P-enriched treatments, reaching 20.1 and 36.6 ×10^3^ cell mL^−1^, for UVR+PAR and PAR treatments, respectively (Fig 6A). By contrast, total biomass, significantly decreased under UVR+PAR treatments compared to PAR after P-enrichment. Consequently, a negative synergistic UVR×P effect was exerted on total abundance and biomass, since the P-enrichment unmasked the inhibitory UVR effect on these variables (Table C in S1 File). The picoplanktonic community played the main role in the magnitude of this response of total phytoplanktonic abundance to UVR×P (Fig 6B). Among microphytoplankton groups, the flagellates underwent a negative synergetic effect UVR×P both in abundance (Fig 6C) and in biomass (Table 2).

Taxonomic composition of the phytoplankton communities at the end of the incubation time are shown in Table 2. In the non-enriched treatment, microphytoplankton biomass groups increased whereas picoeukaryotes and *Synechococcus* sp. biomass declined regardless of the light treatments when compared with initial conditions. P-enrichment did not promote an increase in microphytoplankton biomass, and flagellate biomass significantly decreased in UVR and P-enriched treatment (UVR×P p-value < 0.01) (Table 2). However, the response of picoplankton to P-enrichment was the opposite. Picoeukaryotes and *Synechococcu*s sp. increased in both light treatments after P-enrichment. UVR×P exerted a significantly negative synergistic effect (UVR×P p-value < 0.01) on both groups since P enrichment triggered the inhibitory UVR effect.

## Discussion

In this study, we evaluate, for the first time, the phytoplanktonic responses to the interactive effects of UVR and P enrichment at different biological organization levels (from the physiological to the community level), in an oligotrophic P-limited Mediterranean area. Furthermore, in our experimental approach, we simulated the P enrichment provided by current Saharan dust loads in the western Mediterranean Sea [47, 48], which constitute one of the consequences of global change at the regional scale [3]. Under these conditions, we measured the phytoplanktonic responses to P enrichment over a realistic time scale similar to previously reported phytoplankton blooms in this area of the Mediterranean Sea [71, 72]. This approach provides a framework for unravelling the mechanisms that enable algae to tolerate UVR stress. (attenuation) and at the community level (unmasking) to UVR effect after P-enrichment.

### Tolerance to UVR stress

The C expenditure by respiration, which has been proposed as an indicator of physiological stress, results from catabolic pathways developed to maintain the cell functionality of primary producers under UVR [7]. Our results evidence that UVR increased the phytoplanktonic respiration rate under nutrient ambient conditions. The respiratory electron-transfer chain generates oxygen-free radicals, which increase under photoinhibitory conditions, resulting in the accumulation of ROS [73, 15, 16], an indicator of oxidative stress. Although enzymatic antioxidant activities were not measured in our experiment, the absence of violaxanthin or the decrease in the D_t_:(D_t_+D_d_) ratio, pigments related to the xanthophyll cycle, involved in the thermal dissipation of excess light [74], could partly support the idea of the existence of metabolic stress. Nevertheless, depressed Dd de-epoxidation might also imply an active photoprotective response to UVR exposure via enhanced diadinoxanthin synthesis [75], helping to alleviate the UVR stress, as found in species of bacillariophyceae, haptophyceae or dinophyceae over the time scale of our experiment. In addition, the lack of negative UVR effect on Chl pigments, PP_M_, microphytoplanktonic or picoeukaryotic abundance and biomass could be determined by the start-up of repair mechanisms. Cells reportedly boost the RNA (a P-rich biomolecule) content under UVR to activate the expression of genes related to repair proteins or to provide metabolites needed for cell repair (e.g. ATP) [76, 77, 16]. The start-up of repair mechanisms could be guaranteed by the increase in sestonic P found here (further discussion below). Consequently, the increase in respiration rates could reflect energy costs related to the repair of cellular components damaged by UVR [78, 79]. These above-described mechanisms could operate together, helping to explain that increased oxidative stress did not transfer as damage to the microphytoplankton variables studied. Nevertheless, the metabolic stress generated (increased ROS) was transferred to picoplanktonic C-incorporation and *Synechococcus* sp. abundance and biomass. This result agrees with previous findings showing that eukaryotic phytoplankton have a higher photoacclimation potential than do picoprokaryotic species when both undergo identical experimental light conditions in oligotrophic marine areas [80, 81].

Another less specific mechanism that underlies the eukaryotic phytoplankton acclimation may be the improvement in the cell-quota nutrient shown by the sestonic fraction under UVR, and the decrease in their stoichiometric ratios. The lower C:P ratio under UVR could be attributed either to C-losses (greater respiration or C excretion), or to increased sestonic P content. Our findings imply that the C:P (and N:P) ratios declined under UVR due mainly to greater sestonic P content, since the sestonic C content increased under UVR.

Therefore, the next key question is to ascertain which processes can be related to the increase in the sestonic P content. On the one hand, our results showed a decrease in DOC concentration under UVR in the non-enriched treatment. The lack of increase in bacterial production in our experiment [82] precludes considering the bacterial consumption of DOC as a major mechanism responsible for the observed DOC decrease. Such a drop in the DOC concentration might indicate the UVR-induced photolysis of organic matter, which could actually increase P availability for phytoplankton [83]. On the other hand, our results show that the greater sestonic P per cell was related to surge in enzymatic hydrolysis due to APA, because of the significant increase of APA_EX_ under UVR. Similar UVR effects increasing dissolved APA activity values have been described in freshwater ecosystems [30, 31], where the APA values for different fractions were in the same range as in the present study. Thus, cells exposed to UVR may stimulate P uptake, boosting the sestonic P content. Our results suggest that microplankton phytoplankton is acclimated to UVR, due to induced extracellular APA by UVR. We propose that this mechanism could guarantee P acquisition in P-limited marine ecosystems, where the autotrophic fractions could have a competitive advantage over bacteria to take up inorganic P [84].

### Interactive UVR x P effect

The growth of phytoplankton was stimulated by P addition. Thus, UVR-acclimated phytoplankton (see above) that can use UVA radiation through carotenoids (accessory pigments) as an energy source for photosynthesis [21] might be expected to enhance their growth after a P pulse, and even a positive synergistic interactive effect. This response was found only in ETR_max_ and photosynthetic efficiency (ɑ_ETR_). By contrast, the UVR×P interactive effect was antagonistic on picoplanktonic C-incorporation (PP_P_) and negatively synergetic on other variables such as PP_M_, productivity, and abundance of both size fractions. Therefore, an inhibitory effect of UVR was unmasked after P enrichment.

The finding that UVR×P on picoplanktonic production was antagonistic could be the result, described for these organisms, of an alternative electron flow to O_2_, which extracts electrons from the intersystem electron-transport chain, prior to photosystem I [85]. This pathway alleviates excessive photosystem II excitation pressure that could occur after P enrichment.

The negative synergistic UVR×P effect reported for most of the variables suggests that co-limitation is exerted by high UVR and low nutrient levels on the C metabolism and growth of phytoplankton, because only the removal of both stress factors led to the highest stimulation [35, 20]. The harmful UVR effect after P-enrichment has been widely reported in oligotrophic freshwater ecosystems on phytoplankton abundance [35, 86, 87, 20] and for C fixation (PP) and productivity [20,31]. There are several mechanisms that could alter the coupling of the PSII function, estimated using chlorophyll fluorescence, with photosynthetic carbon fixation, such as photorespiration [88], chlororespiration via a plastid terminal oxidase (PTOX) [85, 89] and the Mehler reaction [90].The lack of increased ROS precludes considering a direct photoreduction of O_2_ by thylakoids, known as the Mehler reaction [91, 92], as a mechanism to explain the decoupling between electron transfer and carbon fixation under UVR and P-enriched conditions. Based on our findings of non-increase in R under UVR after P enrichment, we can rule out a higher carbon loss by respiration, as has been recently proposed [93, 94]. Unfortunately, we did not measure photorespiration, a key process which could account for the mismatch between ETR_max_ and C incorporation into biomass, because the photorespiration can imply a loss of up to 25% of the C fixed in photosynthetic processes [95]. Nevertheless, we may speculate that the excreted C (not increased under UVR after P-enrichment) failed to eliminate phosophoglycolate (a by-product of photorespiration), which inflicts damage similar to that of ROS [96, 97]. Furthermore, this unmasking effect on phytoplanktonic abundance could be the result of growth stimulated by enrichment with limiting nutrients, thereby inducing higher rates of DNA synthesis. This may result in a greater propensity to UVR-induced DNA damage, making the effects of UVR on cell division variables more evident after P enrichment [98].

On the other hand, our results demonstrate that picoprokaryotes in the near-surface layers of the Alborán Sea may be severely affected by exposure to ambient levels of UVR, limiting their growth despite their competitive advantage under P-enriched conditions. The *Synechococcus* sp. populations underwent higher inhibitory UVR effects than did *Prochlorococcus* sp.. These results are contrary to those of Sommaruga et al. [99] and LLabré et al. [18] for the north-western Mediterranean Sea (NWS). It is remarkable that the abundance of picoprokaryotes was lower (by one order of magnitude) in Alborán Sea than in NWS [99,100]. The higher sensitivity of *Synechococcus* sp. even after a P pulse suggests that the levels of photoprotection and repair systems could be insufficient to repair the cell damage induced by solar radiation.

## Concluding Remarks

Based on our findings, we propose that (1) the main mechanism of autotrophic eukaryote tolerance to UVR is the increase in P content mediated by (i) a direct stimulatory effect of UVR on dissolved fraction of extracellular APA reinforced by greater DOC-photolysis, (ii) an increase in P uptake under UVR and an improvement of the P cell-quota, allowing the repair of cell components damaged by sublethal levels of UVR; (2) the mechanism involved in the unmasking effect of UVR after P enrichment may be the photodamage caused by excessive electron flux with the activation of photosynthetic electron transport, in the absence of an efficient C-release mechanism(by eliminating the phosophoglycolate) to dissipate the reducing power of photosynthetic electron transport. This photodamage would explain the mismatch between the synergistic positive UVR×P effect on photosynthetic variables and the synergistic negative UVR×P effect on C incorporation and productivity that constrains the growth of the phytoplankton community.

## Supporting Information

S1 File Results of the two-way ANOVA of the interaction of UVR and P-enrichment. Interactive effect on respiration, reactive oxygen species, total and dissolved alkaline phosphatase and dissolved organic carbon **(Table A)**. Interactive effect on C, P and N cell-quota, particulate organic carbon, particulate organic nitrogen, particulate organic phosphorus, C:P, C:N and N:P ratios **(Table B)**. Interactive effect on maximal electron transport rate, photosynthetic efficiency, Dd de-epoxidation; microphytoplanktonic and, picophytoplanktonic carbon incorporation, percentage excreted organic carbon, productivity and total abundance **(Table C)**. F values and significance levels (p) are shown, numbers in bold indicate, p-value < 0.05. R: respiration; ROS: reactive oxygen species; APA_T_:t otal alkaline phosphatase activity;APA_EX_: dissolved alkaline phosphatase activity; DOC: dissolved organic carbon. POC: particulate organic carbon; PON: particulate organic nitrogen; POP: particulate organic phosphorus ETR_max_: maximal electron transport rate; αETR: photosynthetic efficiency; Dt:Dt+Dd: Dd de-epoxidation; PP_M_: microphytoplanktonic carbon incorporation; PP_P_: picophytoplanktonic carbon incorporation;%EOC: Percentage excreted organic carbon.
(PDF)
