# Supplementary material for: Interactive Effect of UVR and Phosphorus on the Coastal Phytoplankton Community of the Western Mediterranean Sea: Unravelling Eco-Physiological Mechanisms
Source: PLoS One. 2015 Nov 23;10(11):e0142987. doi: 10.1371/journal.pone.0142987 (PMC4658109; doi:10.1371/journal.pone.0142987)
Supplement: S1 File — Interactive effect on respiration, reactive oxygen species, total and dissolved alkaline phosphatase and dissolved organic carbon (Table A). Interactive effect on C, P and N cell-quota, particulate organic carbon, particulate organic nitrogen, particulate organic phosphorus, C:P, C:N and N:P ratios (Table B). Interactive effect on maximal electron transport rate, photosynthetic efficiency, Dd de-epoxidation; microphytoplanktonic and, picophytoplanktonic carbon incorporation, percentage excreted organic carbon, productivity and total abundance (Table C). F values and significance levels (p) are shown, numbers in bold indicate, p-value < 0.05. R: respiration; ROS: reactive oxygen species; APAT:t otal alkaline phosphatase activity;APAEX: dissolved alkaline phosphatase activity; DOC: dissolved organic carbon. POC: particulate organic carbon; PON: particulate organic nitrogen; POP: particulate organic phosphorus ETRmax: maximal electron transport rate; αETR: photosynthetic efficiency; Dt:Dt+Dd: Dd de-epoxidation; PPM: microphytoplanktonic carbon incorporation; PPP: picophytoplanktonic carbon incorporation;%EOC: Percentage excreted organic carbon. (PDF) [file pone.0142987.s001.pdf]

Table A

.

Variable	Effect	F-value	P-value
R	UVR	21.21	**0.002**
	P	16.66	**0.004**
	UVR×P	10.44	**0.012**
ROS	UVR	82.56	**0.000**
	P	28.33	**0.001**
	UVR×P	83.29	**0.000**
APA_T	UVR	13.01	**0.007**
	P	275.73	**0.000**
	UVR×P	0.03	0.867
APA_{EX}	UVR	41.16	**0.000**
	P	159.94	**0.000**
	UVR×P	1.16	0.313
DOC	UVR	167.416	**0.000**
	P	282.439	**0.000**
	UVR×P	380.647	**0.000**

Table B

Variable	Effect	F-value	P-value
C cell quota	UVR	51.77	**0.000**
	P	186.75	**0.000**
	UVR×P	5.48	**0.047**
N cell quota	UVR	18.55	**0.003**
	P	41.86	**0.000**
	UVR×P	2.52	0.151
P cell quota	UVR	37.87	**0.000**
	P	4.22	0.074
	UVR×P	0.09	0.771
POC	UVR	16.05	**0.004**
	P	9.15	**0.016**
	UVR×P	2.89	0.128
PON	UVR	13.60	**0.006**
	P	3.88	0.084
	UVR×P	1.14	0.318
POP	UVR	8.86	**0.018**
	P	45.41	**0.000**
	UVR×P	0.00	0.946
C:P ratio	UVR	10.27	**0.013**
	P	80.51	**0.000**
	UVR× P	3.48	0.099
C:N ratio	UVR	4.00	0.081
	P	0.19	0.677
	UVR×P	0.01	0.924
N:P ratio	UVR	5.43	**0.048**
	P	65.83	**0.000**
	UVR×P	3.68	0.091

Table C

Variable	Effect	F-value	P-value
ETR_{max}	UVR	18.83	**0.002**
	P	152.49	**0.000**
	UVR×P	13.13	**0.007**
α_{ETR}	UVR	10.44	**0.012**
	P	129.10	**0.000**
	UVR×P	12.62	**0.007**
$D_t:(D_t+D_d)$	UVR	1.50	**0.255**
	P	4.84	**0.059**
	UVR×P	7.56	**0.025**
PP_M	UVR	14.58	**0.005**
	P	360.89	**0.000**
	UVR×P	60.70	**0.000**
PP_P	UVR	7.52	**0.025**
	P	206.42	**0.000**
	UVR×P	8.43	**0.020**
%EOC	UVR	40.83	**0.000**
	P	18.71	**0.003**
	UVR×P	48.05	**0.000**
Productivity	UVR	16.44	**0.004**
	P	76.59	**0.000**
	UVR×P	8.78	**0.040**
Total Abundance	UVR	154.49	**0.000**
	P	1201.83	**0.000**
	UVR×P	117.56	**0.000**
